# Depressive symptoms and health-related quality of life in critical COVID-19 survivors: Preliminary results of 1-year follow-up

**DOI:** 10.1192/j.eurpsy.2022.793

**Published:** 2022-09-01

**Authors:** S. Martins, L. Fontes, A.R. Ferreira, J. Fernandes, T. Vieira, N. Reis, A. Braga, I. Coimbra, J.A. Paiva, L. Fernandes

**Affiliations:** 1 Faculty of Medicine - University Porto, Department Of Clinical Neuroscience And Mental Health And Center for health technology and services research (cintesis), Porto, Portugal; 2 Centro Hospitalar Universitário São João (CHUSJ), Intensive Care Medicine Department, Porto, Portugal; 3 Faculty of Medicine - University Porto, Department Of Medicine, Porto, Portugal; 4 Psychiatry Service, Centro Hospitalar Universitário De São João, Porto, Portugal

**Keywords:** Depression, Covid-19, health-related quality of life

## Abstract

**Introduction:**

A higher risk of mental health consequences in critical COVID-19 patients is expected due to several reasons, including prolonged mechanical ventilation with exposure to high sedation. In this context, post-discharge depression has been reported in previous COVID-19 studies, with a profound impact on patients’ health-related quality of life (HRQoL).

**Objectives:**

To identify depressive symptoms in COVID-19 survivors 1-year after hospital discharge and to analyse its association with HRQoL.

**Methods:**

As part of the longitudinal MAPA project, this study enrolled critical COVID-19 patients admitted in the Intensive Care Medicine Department of a University Hospital (March-May 2020). Participants were assessed through telephone by an intensive care nurse and a psychologist, with the Patient Health Questionnaire (PHQ-9) (depressive symptoms), EuroQol five-dimension five-level questionnaire (EQ-5D-5L) and EQ-Visual Analogue Scale (EQ-VAS) (global health status patient record).

**Results:**

A sample of 55 survivors (median age=66 years; 69% males) were included, with 20% showing depressive symptoms. Pain/discomfort (67%) and anxiety/depression (67%) were the most EQ-5D-5L domains reported. Survivors scoring for depression had more problems in all HRQoL areas (mobility:91%vs.48%, p=0.015; self-care:64%vs.27%, p=0.035; usual activities:91%vs.50%, p=0.017; pain/discomfort:100%vs.59%, p=0.010; anxiety/depression:100%vs.59%, p=0.010). Moreover, they had a lower EQ-VAS median, corresponding a worse self-perception of health status (50vs.80, p=0.010).

**Conclusions:**

Even after 1-year, a significant proportion of survivors presented depressive symptoms with repercussions in all HRQoL dimensions and association with worse self-perception of global quality of life. Taking this in mind, early screening and treatment of depression in COVID-19 survivors will be crucial, minimizing its impact on quality of life.

**Disclosure:**

No significant relationships.

